# TAO-DFT with the Polarizable Continuum Model

**DOI:** 10.3390/nano13101593

**Published:** 2023-05-10

**Authors:** Sonai Seenithurai, Jeng-Da Chai

**Affiliations:** 1 Department of Physics, National Taiwan University, Taipei 10617, Taiwan; seenithurai@gmail.com; 2 Center for Theoretical Physics and Center for Quantum Science and Engineering, National Taiwan University, Taipei 10617, Taiwan; 3 Physics Division, National Center for Theoretical Sciences, Taipei 10617, Taiwan

**Keywords:** TAO-DFT, polarizable continuum model, multi-reference character, solvation effects, nanomolecules, linear acenes

## Abstract

For the ground-state properties of gas-phase nanomolecules with multi-reference character, thermally assisted occupation (TAO) density functional theory (DFT) has recently been found to outperform the widely used Kohn–Sham DFT when traditional exchange-correlation energy functionals are employed. Aiming to explore solvation effects on the ground-state properties of nanomolecules with multi-reference character at a minimal computational cost, we combined TAO-DFT with the PCM (polarizable continuum model). In order to show its usefulness, TAO-DFT-based PCM (TAO-PCM) was used to predict the electronic properties of linear acenes in three different solvents (toluene, chlorobenzene, and water). According to TAO-PCM, in the presence of these solvents, the smaller acenes should have nonradical character, and the larger ones should have increasing polyradical character, revealing striking similarities to the past findings in the gas phase.

## 1. Introduction

Electronic structure methods are commonly used to compute the properties of gas-phase molecules. Nonetheless, the properties of molecules in solutions can be very different from those of gas-phase molecules. Therefore, it is important to realize the effects of solvents on the properties of solute molecules, especially when polar solvents are involved. While it is possible to adopt explicit solvent models (wherein solvation effects are taken into consideration by explicitly including the molecular details of each solvent molecule) [1,2,3], the resulting electronic structure calculations can, however, be computationally intractable in many applications.

In order to circumvent this difficulty, several implicit solvent models have been devised to study the properties of molecules in polarizable solvents over the past few decades. Among them, the Kirkwood–Onsager model [4,5,6] is perhaps the simplest one. However, the Kirkwood–Onsager (KO) model is not appropriate for non-spherical solute molecules because of its underlying assumptions.

In order to resolve the shortcomings of the KO model, recently developed implicit solvent models, such as the PCM (polarizable continuum model) [1,2,3,7,8,9,10,11,12,13,14,15,16,17,18,19,20,21,22], have become popular due to their reasonably accurate description of solvation effects at a low computational cost. According to the PCM, the solvent is modeled as a polarizable continuum (i.e., a homogeneous dielectric medium rather than individual molecules), and the solute is placed inside a molecule-shaped cavity surrounded by the continuum solvent [1,2,3]; typically, the solute is treated in a quantum-mechanical manner with an electronic structure method, and the solvation effect is modeled implicitly with a PCM. In the past few years, various types of PCMs, such as the dielectric version of PCM (D-PCM) [7], COSMO (conductor-like screening model) [8], COSMO-RS (COSMO for real solvents) [9], GCOSMO (generalized COSMO) [10], C-PCM (conductor-like PCM) [11], IEF-PCM (integral equation formalism of the PCM) [12,13,14,15], SS(V)PE (surface and simulation of volume polarization for electrostatics) model [16,17,18,19,20], and many others [21,22], have been developed to properly describe solvation effects.

On the other hand, among the presently available electronic structure methods, Kohn–Sham (KS) density functional theory (DFT) [23] has been regarded as the method of choice for predicting the ground-state (GS) properties of nanomolecules in the gas phase. Accordingly, KS-DFT-based PCM (KS-PCM) [1,2,3] has been widely employed for exploring the properties of nanomolecules in solutions. However, in KS-DFT, the exact XC (exchange-correlation) energy functional remains unavailable, and the commonly used XC functionals are approximate and may suffer from a number of serious issues [24,25].

For instance, KS-DFT with the commonly used local, semilocal, and hybrid XC energy functionals may lead to unreliable results for the GS properties of gas-phase molecules with multi-reference (MR) character. Therefore, it can be anticipated that the corresponding KS-PCM can also lead to incorrect solvation effects on the ground-state properties of these molecules. Typically, accurate MR electronic structure methods [26,27,28,29,30,31,32,33,34,35] are required for studying gas-phase molecules with MR character. Nonetheless, these MR methods and related PCMs [36] are computationally infeasible for nanomolecules in the gas phase and solution phase, respectively, limiting their applications only to very small molecules.

In order to predict the GS properties of gas-phase nanomolecules with MR character, thermally assisted occupation (TAO) DFT [37], i.e., a DFT using the fractionally occupied TAO-orbitals, has recently been proposed. Since the TOONs (TAO-orbital occupation numbers) are directly produced by the FD (Fermi–Dirac) distribution with some fictitious temperature θ, TAO-DFT, which is as efficient as KS-DFT in computational expense, appears to be a promising electronic structure method for predicting gas-phase nanomolecules with MR character. Furthermore, the conventional local [37], semilocal [38], and hybrid [39] XC energy functionals (e.g., those defined in KS-DFT) can also be used in TAO-DFT. In addition, several TAO-DFT-extensions [40,41,42,43,44] have recently been developed as well. Over the last few years, TAO-DFT has been used to predict the electronic [45,46,47,48,49,50,51,52,53,54,55,56], hydrogen storage [47], and spectroscopic [43,57,58] properties of gas-phase nanomolecules with MR character, outperforming KS-DFT with common XC energy functionals.

It is worth mentioning that some interesting electronic structure methods [59,60,61,62,63] closely related to TAO-DFT have also been developed in recent years. Moreover, a few recent studies [64,65,66,67] on the GS properties of electronic systems at absolute zero have been actually performed using TAO-DFT without the θ-dependent energy functional [37] (i.e., an approximate TAO-DFT approach) at some fictitious temperature θ, which should not be confused with the Mermin–Kohn–Sham method (also called finite-temperature density functional theory (FT-DFT)) [23,68] at some finite electronic temperature due to their distinctly different physical meanings (e.g., see Refs. [37,44,53] for further discussion).

**Figure 1:**
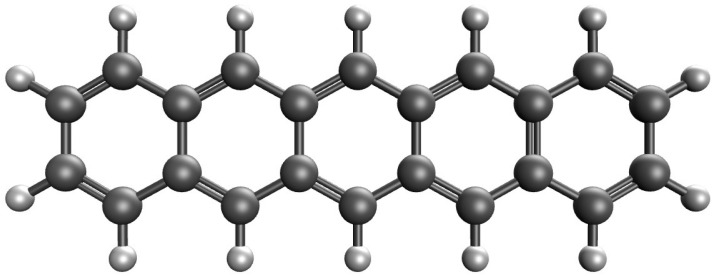
Geometry of 5-acene (i.e., the solute), containing five aromatic rings.

Accordingly, here, we combined TAO-DFT with the PCM to model solvation effects on the GS properties of nanomolecules with MR character. The resulting TAO-DFT-based PCM, denoted as TAO-PCM, is expected to improve on the widely used KS-PCM for the properties of solute molecules with MR character when solvents are involved. In large part, this is because the MR character of solute molecules is inherently quantum-mechanical, which should be better described by TAO-DFT than by KS-DFT when traditional XC energy functionals are employed. In order to show its usefulness, we also employed TAO-PCM to explore the effects of solvents on the electronic properties (the singlet–triplet gap, vertical electron affinity/ionization potential, fundamental gap, etc.) of *n*-acene (i.e., a linear acene with *n* aromatic rings, e.g., see Figure 1) in three different solvents (toluene, chlorobenzene, and water). Since the larger *n*-acenes in the gas phase have recently been found to exhibit MR character in their ground states [27,29,37,39,43,44,45], the larger *n*-acenes in solvents are also expected to exhibit MR character (as will been shown later). Furthermore, some shortcomings of KS-PCM related to the issues of MR character are found to be greatly resolved by TAO-PCM.

## 2. TAO-PCM

Consider a solute molecule (with *N* electrons) surrounded by solvent molecules. In the PCM [1,2,3,7,8,9,10,11,12,13,14,15,16,17,18,19,20,21,22], the solvent is modeled as a homogeneous dielectric medium (i.e., a polarizable continuum, rather than individual molecules), and a molecule-shaped cavity, which separates the solute and the continuum solvent, is constructed (e.g., using a union of atom-centered spheres with the scaled van der Waals (vdW) radii), wherein the dielectric constant is set equal to 1 inside the cavity (as in vacuum), and is set equal to ϵ (i.e., the bulk value of the considered solvent) outside the cavity.

In TAO-PCM, the solute is treated in a quantum-mechanical manner with TAO-DFT [37], and the solvation effect is modeled implicitly with the PCM. Specifically, in the presence of the continuum solvent, the TAO-PCM solute free energy $G_{\mathrm{TAO}-\mathrm{PCM}}\left[\rho \right]$ is a functional of the solute electron density ρ(r) (atomic units were adopted throughout this work):

(1) $$G_{\mathrm{TAO}-\mathrm{PCM}}\left[\rho \right]=E_{\mathrm{TAO}}\left[\rho \right]+V_{\mathrm{NN}}+G_{\mathrm{PCM}}\left[\rho \right],$$

where $E_{\mathrm{TAO}}\left[\rho \right]$ (e.g., see Equation (27) of Ref. [37]) is the gas-phase solute electronic energy in TAO-DFT, $V_{\mathrm{NN}}$ is the solute nuclear repulsion energy, and $G_{\mathrm{PCM}}\left[\rho \right]$ (e.g., see Equation (14) of Ref. [21]) is the solute–solvent interaction free energy in the PCM.

In order to obtain the solute electron density ρ(r), the TAO-PCM self-consistent equations are given by

(2) $$\left\{-\frac{1}{2}\nabla^{2}+v_{\mathrm{TAO}}\left(r\right)+v_{\mathrm{PCM}}\left(r\right)\right\}\psi_{i}\left(r\right)=\epsilon_{i}\psi_{i}\left(r\right).$$

Here, $v_{\mathrm{TAO}}\left(r\right)$ (e.g., see Equation (18) of Ref. [37]) is the gas-phase solute effective potential in TAO-DFT, $v_{\mathrm{PCM}}\left(r\right)=\delta G_{\mathrm{PCM}}\left[\rho \right]/\delta \rho \left(r\right)$ (e.g., see Equation (17) of Ref. [21]) is the reaction potential in the PCM, and the solute electron density ρ(r) is expressed as

(3) $$\rho \left(r\right)=\sum_{i}f_{i}{\left|\psi_{i}\left(r\right)\right|}^{2},$$

where $f_{i}$ (the occupation number associated with the *i*-th TAO-orbital $\psi_{i}\left(r\right)$) is generated by the FD distribution (with some fictitious temperature θ):

(4) $$f_{i}={\left\{1+\exp\left[\left(\epsilon_{i}-\mu \right)/\theta \right]\right\}}^{-1}$$

satisfying the constraints $0\leq f_{i}\leq 1$ and $\sum_{i}f_{i}=N$, with $\epsilon_{i}$ being the energy associated with the *i*-th TAO-orbital $\psi_{i}\left(r\right)$, and μ being the chemical potential.

For the θ=0 case, as TAO-DFT reduces to KS-DFT, TAO-PCM reduces to KS-PCM. Note also that the PCM component $G_{\mathrm{PCM}}\left[\rho \right]$, as well as $v_{\mathrm{PCM}}\left(r\right)$, which depends only on the solvent dielectric constant ϵ and the solute (electronic and nuclear) charge density [21], has the same expression in TAO-PCM as in KS-PCM. In addition, for an isolated solute molecule (i.e., ϵ=1), as $G_{\mathrm{PCM}}\left[\rho \right]=0$ and $v_{\mathrm{PCM}}\left(r\right)=0$, TAO-PCM reduces to TAO-DFT, and KS-PCM reduces to KS-DFT.

## 3. Computational Details

We performed all calculations using the 6-31G(d) basis set with the software package of Q-Chem 4.4 [69]. As mentioned previously, in TAO-PCM, TAO-DFT is adopted for the electronic structure calculations, and the PCM is adopted for the modeling of solvent effects. In this study, for the TAO-DFT part, we adopted TAO-LDA [37], which is TAO-DFT with the local density approximation (LDA) XC and θ-dependent energy functionals (with θ = 7 mhartree (i.e., the suggested fictitious temperature)). Specifically, we used the PW92 parametrization [70] of the LDA correlation energy functional and the LDA θ-dependent energy functional [37] obtained with the Perrot parametrization [71] of the LDA noninteracting kinetic free energy functional. While other θ values are also possible for TAO-LDA (which could lead to slightly different electronic properties) [37,40,44], TAO-LDA (θ = 7 mhartree) has recently been shown to yield a qualitatively similar MR character of various alternant polycyclic aromatic hydrocarbons (e.g., *n*-acenes) [46] when compared with an accurate MR electronic structure method [28]. In addition, TAO-LDA (θ = 7 mhartree) has been commonly employed to study the electronic properties of gas-phase nanomolecules with MR character [45,48,49,50,51,52,53]. Because of its decent compromise between accuracy and efficiency, in this TAO-PCM study, we adopted TAO-LDA (θ = 7 mhartree) for the TAO-DFT part.

On the other hand, for the PCM part, we adopted C-PCM [11], which has been a popular PCM due to its simplicity in formalism and implementation as well as its decent balance between efficiency and accuracy. Note also that C-PCM is an approximation of COSMO [8] and can also be regarded as the high-dielectric limit associated with IEF-PCM [12,13,14,15]. Regarding the construction and discretization of a solute cavity surface, the solute cavity surface [3] was formed using a union of atom-centered spheres with their radii being 1.2 times the atomic vdW radii [72,73,74]. In addition, the SWIG (switching/Gaussian) approach was adopted for smooth cavity discretization [75,76], with 194 Lebedev grid points per atomic sphere. Furthermore, for the solvent dielectric constant ϵ, we adopted the Q-Chem default value, i.e., 2.38 for toluene, 5.62 for chlorobenzene, and 78.39 for water.

For comparison with the results of TAO-PCM (i.e., TAO-LDA/C-PCM), we also reveal some results obtained with the corresponding KS-PCM (i.e., KS-LDA/C-PCM), wherein KS-LDA (i.e., KS-DFT with the LDA XC energy functional, which is the same as TAO-LDA with θ=0) was used for the electronic structure calculations, and C-PCM was used for the modeling of solvent effects.

## 4. Results and Discussion

### 4.1. Singlet-Triplet Energy Gap

**Figure 2:**
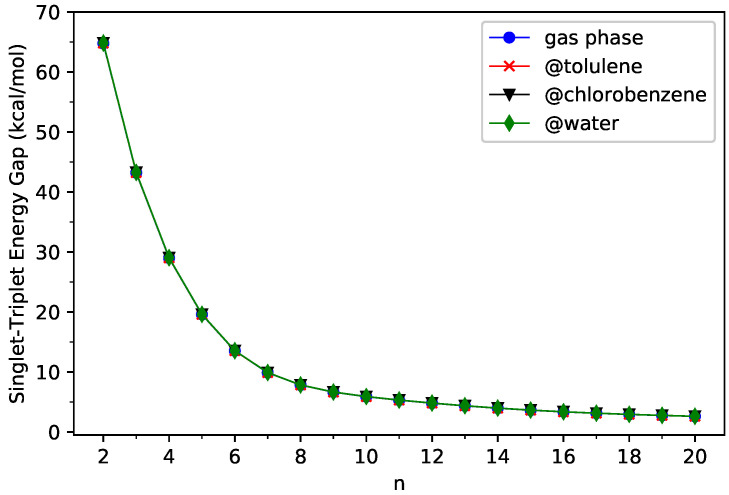
Singlet–triplet energy gap of *n*-acene in three different solvents (toluene, chlorobenzene, and water) and in the gas phase, obtained from spin-unrestricted TAO-PCM (i.e., TAO-LDA/C-PCM).

**Figure 3:**
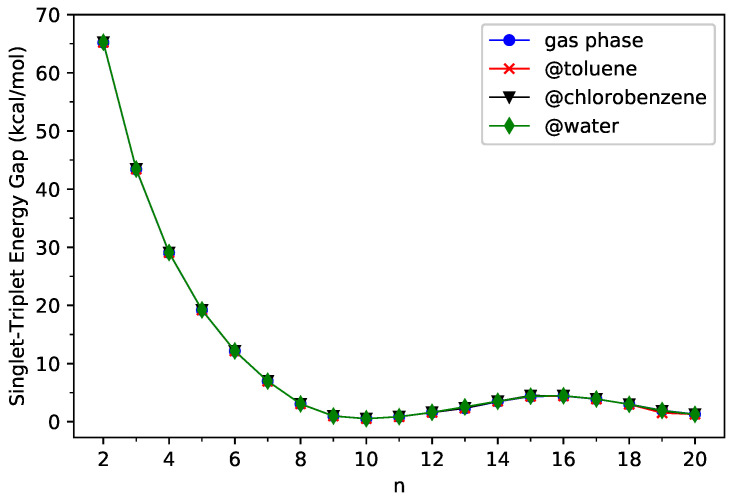
Singlet–triplet energy gap of *n*-acene in three different solvents (toluene, chlorobenzene, and water) and in the gas phase, obtained from spin-unrestricted KS-PCM (i.e., KS-LDA/C-PCM).

A singlet–triplet energy gap (ST gap) can provide many insights on chemical processes. In order to obtain the ST gap of *n*-acene (i.e., the solute) in the continuum solvent, we fully optimized the geometries of the lowest singlet and triplet states of *n*-acene in the continuum solvent using spin-unrestricted TAO-PCM (i.e., TAO-LDA/C-PCM), and computed the ST gap of *n*-acene in the continuum solvent using

(5) $$E_{\mathrm{ST}}=G_{\mathrm{UT}}-G_{\mathrm{US}},$$

where $G_{\mathrm{US}}$ and $G_{\mathrm{UT}}$ are the spin-unrestricted TAO-PCM free energies (given by Equation (1)) associated with the lowest singlet and lowest triplet states, respectively, of *n*-acene in the continuum solvent. For comparison purposes, we additionally report the results of the corresponding KS-PCM (i.e., KS-LDA/C-PCM). As presented in Figure 2 and Figure 3 (see Tables S1 and S2 in the SI (Supplementary Information) for additional information), the ST gaps of *n*-acenes in three different solvents (toluene, chlorobenzene, and water) are essentially the same as those of *n*-acenes in the gas phase, indicating that the solvation effects play insignificant roles here. In addition, for all the cases investigated, *n*-acenes in different media possess singlet ground states. As molecules with MR character are commonly characterized by small ST gaps, similar to the gas-phase situations [37,38,39,43,44,45], in the three solvents, the larger *n*-acenes, which have smaller ST gaps, are expected to have more significant MR character than the smaller *n*-acenes.

On the other hand, the ST gap of TAO-PCM decreases with the size of *n*-acene in a monotonic manner, but the ST gap of KS-PCM exhibits an unexpected increase beyond 10-acene. Therefore, we also explored the potential causes of such discrepancies by examining the expectation values of ${\hat{S}}^{2}$ (i.e., the total spin-squared operator) associated with the lowest singlet and lowest triplet states of *n*-acene in the continuum solvent, calculated by spin-unrestricted KS-PCM (see Table 1 and Table 2). For the KS-PCM wavefunction with an artificial mixing of several spin-states (i.e., the so-called spin contamination), the respective $\langle {\hat{S}}^{2}\rangle$ value will be larger than the exact $\langle {\hat{S}}^{2}\rangle$ value (i.e., 2 for the lowest triplet state and 0 for the lowest singlet state) [49,53,77]. Accordingly, the difference between the $\langle {\hat{S}}^{2}\rangle$ value of KS-PCM and the exact $\langle {\hat{S}}^{2}\rangle$ value can be regarded as a measure of the degree of spin contamination in the spin-unrestricted KS-PCM wavefunction. As shown, for the smaller *n*-acenes, the $\langle {\hat{S}}^{2}\rangle$ values of KS-PCM are very close to the exact $\langle {\hat{S}}^{2}\rangle$ values, implying that the lowest singlet and triplet states of the smaller *n*-acenes in different media, obtained from spin-unrestricted KS-PCM, essentially have no spin contamination. Nonetheless, for the lowest singlet states of some larger *n*-acenes (e.g., *n* = 9–12), the $\langle {\hat{S}}^{2}\rangle$ values of KS-PCM are considerably larger than the exact $\langle {\hat{S}}^{2}\rangle$ values, indicating that the lowest singlet states of some larger *n*-acenes in different media, obtained from spin-unrestricted KS-PCM, are heavily spin-contaminated. Consequently, the larger *n*-acenes can have MR character in the lowest singlet states, and the unexpected increase in the ST gap of KS-PCM beyond 10-acene could simply be an artifact related to spin contamination.

Furthermore, we can also assess the effects of spin contamination in the lowest singlet free energies of *n*-acenes in different media, obtained from spin-unrestricted TAO-PCM and KS-PCM. Because of the spin-symmetry constraint [37,38,39], for the PCM with an exact electronic structure method, the lowest singlet free energy of *n*-acene in the continuum solvent, calculated by the spin-restricted formalism, has to be identical to that calculated by the spin-unrestricted formalism. In order to assess whether this constraint can be obeyed in TAO-PCM, we fully optimized the geometries of the lowest singlet state of *n*-acene in the continuum solvent using spin-restricted and spin-unrestricted TAO-PCM, and subsequently computed the $E_{\mathrm{UR}}$ value (i.e., the difference between the lowest spin-unrestricted and lowest spin-restricted singlet free energies) of *n*-acene in the continuum solvent using

(6) $$E_{\mathrm{UR}}=G_{\mathrm{RS}}-G_{\mathrm{US}},$$

where $G_{\mathrm{RS}}$ and $G_{\mathrm{US}}$ are the TAO-PCM free energies (given by Equation (1)) associated with the lowest singlet state of *n*-acene in the continuum solvent, computed using the spin-restricted and spin-unrestricted formalisms, respectively. For comparison, we also report the corresponding KS-PCM results. As shown in Table 3, the $E_{\mathrm{UR}}$ values of *n*-acenes in the continuum solvent, calculated by KS-PCM, are rather large for some larger *n*-acenes (e.g., $E_{\mathrm{UR}}\geq 3.20$ kcal/mol for 10-acene), yielding unphysical spin-symmetry-breaking results in the corresponding spin-unrestricted KS-PCM calculations. This implies that, in spin-unrestricted KS-PCM, the up-spin electron density can be rather different from the down-spin electron density for the lowest singlet state of larger *n*-acene in the continuum solvent (even when these spin densities are required to be the same due to the spin-symmetry constraint). Consequently, for the lowest singlet state of larger *n*-acene in the continuum solvent, an electronic property (which depends on the spin densities) obtained with spin-unrestricted KS-PCM can be rather different from that obtained with spin-restricted KS-PCM (even when these electronic properties are required to be the same due to the spin-symmetry constraint). Clearly, such an unphysical spin-symmetry-breaking feature of spin-unrestricted KS-PCM is very undesirable. By contrast, the $E_{\mathrm{UR}}$ values of *n*-acenes in the continuum solvent, calculated by TAO-PCM, are essentially zero for all the cases investigated, leading to essentially no unphysical spin-symmetry-breaking results in the corresponding spin-unrestricted TAO-PCM calculations.

In summary, the ST gaps of the larger *n*-acenes in the continuum solvent, computed using KS-PCM, can be severely influenced by spin contamination, seriously degrading the accuracy of KS-PCM in predicting the ST gaps and possibly other electronic properties (e.g., those that depend on the spin densities). As will be shown later, the lowest singlet states (i.e., ground states) of the larger *n*-acenes in different media exhibit an increasing polyradical nature, wherein KS-PCM could lead to unreliable electronic properties. Consequently, we merely present the TAO-PCM results hereafter.

### 4.2. Vertical Electron Affinity/Ionization Potential and Fundamental Gap

For a neutral solute molecule (with *N* electrons) in a continuum solvent, the free energy gained when an electron is added to the neutral solute molecule (without altering the solute geometry) is defined as the vertical electron affinity

(7) $${\mathrm{EA}}_{v}=G_{N}-G_{N+1},$$

the free energy that is required to remove an electron from the neutral solute molecule (without altering the solute geometry) is defined as the vertical ionization potential

(8) $${\mathrm{IP}}_{v}=G_{N-1}-G_{N},$$

and their difference is defined as the fundamental gap [38,39]

(9) $$E_{g}={\mathrm{IP}}_{v}-{\mathrm{EA}}_{v}=G_{N+1}+G_{N-1}-2G_{N}.$$

In this study, $G_{N}$, $G_{N+1}$, and $G_{N-1}$ are the spin-unrestricted TAO-PCM free energies (given by Equation (1)) associated with the neutral, anionic, and cationic states, respectively, of *n*-acene (i.e., the solute) in the continuum solvent.

**Figure 4:**
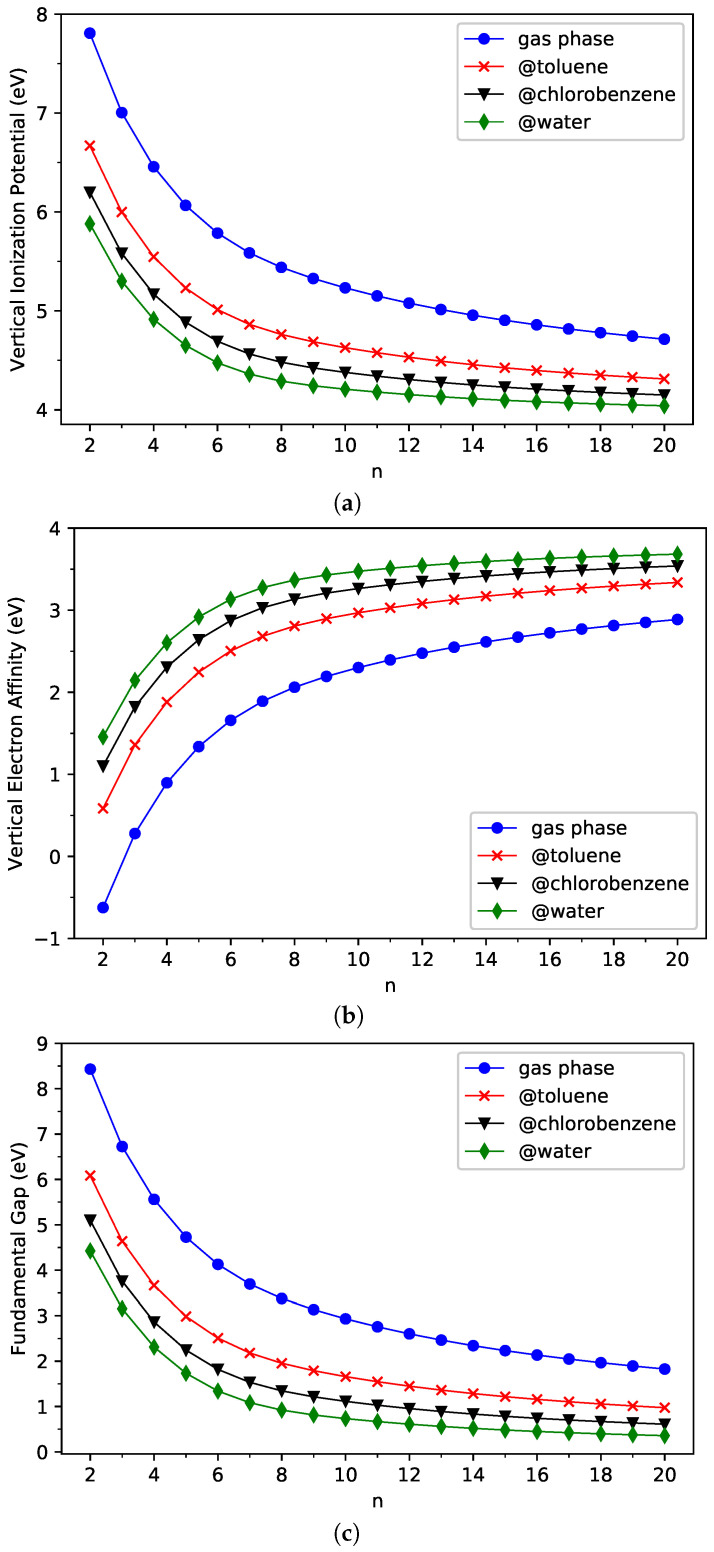
(**a**) Vertical ionization potential, (**b**) vertical electron affinity, and (**c**) fundamental gap for the ground state of *n*-acene in three different solvents (toluene, chlorobenzene, and water) and in the gas phase, obtained from spin-unrestricted TAO-PCM (i.e., TAO-LDA/C-PCM).

Figure 4 (also see Tables S3–S5 in the SI for additional information) presents the ${\mathrm{IP}}_{v}$, ${\mathrm{EA}}_{v}$, and $E_{g}$ of ground-state *n*-acene in three different solvents (toluene, chlorobenzene, and water) and in the gas phase, obtained with spin-unrestricted TAO-PCM (i.e., TAO-LDA/C-PCM). As shown, for each solvent studied, the ${\mathrm{IP}}_{v}$ decreases in a monotonic manner as the size of *n*-acene increases. In addition, for each *n*-acene studied, the ${\mathrm{IP}}_{v}$ decreases as the solvent dielectric constant ϵ increases, indicating that less free energy is needed to remove an electron from *n*-acene in the continuum solvent of larger ϵ. Therefore, the ${\mathrm{IP}}_{v}$ of *n*-acene is the largest in the gas phase (ϵ=1), and is the smallest in water (ϵ=78.39).

A similar but opposite trend is observed from the ${\mathrm{EA}}_{v}$. As the size of *n*-acene increases, the ${\mathrm{EA}}_{v}$ increases in a monotonic manner for each solvent studied. In addition, for each *n*-acene studied, the ${\mathrm{EA}}_{v}$ increases as the solvent dielectric constant ϵ increases, indicating that more free energy is obtained when an electron is added to *n*-acene in the continuum solvent of larger ϵ. Accordingly, the ${\mathrm{EA}}_{v}$ of *n*-acene is the smallest in the gas phase (ϵ=1) and is the largest in water (ϵ=78.39).

Because of the monotonically decreasing nature of ${\mathrm{IP}}_{v}$ and the monotonically increasing nature of ${\mathrm{EA}}_{v}$ with an increasing *n*-acene size, unsurprisingly, for each solvent studied, the $E_{g}$ decreases in a monotonic manner with an increasing *n*. Moreover, for each *n*-acene studied, the $E_{g}$ decreases as the solvent dielectric constant ϵ increases. Consequently, the $E_{g}$ of *n*-acene is the largest in the gas phase (ϵ=1) and is the smallest in water (ϵ=78.39).

In short, our study suggests that the solvation effects are rather important in the ${\mathrm{IP}}_{v}$, ${\mathrm{EA}}_{v}$, and $E_{g}$ values of ground-state *n*-acenes since their values can be easily tuned by changing solvents. This highlights the significance of TAO-PCM in the modeling of solvent effects on the GS properties of nanomolecules with MR character.

### 4.3. Symmetrized von Neumann Entropy

Similar to the TOONs in TAO-DFT [37,38,39,44], the TOONs in TAO-PCM can be approximately viewed as the NOONs (natural orbital occupation numbers) [78] for the ground state of a solute molecule in the continuum solvent. Hence, the strength of the MR character of ground-state *n*-acene in the continuum solvent can be estimated using the corresponding value of $S_{\mathrm{vN}}$ (symmetrized von Neumann entropy) [38,39,79]:

(10) $$S_{\mathrm{vN}}=-\frac{1}{2}\sum_{i}\left\{f_{i}\;\ln\left(f_{i}\right)+\left(1-f_{i}\right)\;\ln\left(1-f_{i}\right)\right\},$$

where $\left\{f_{i}\right\}$ are the TOONs (see Equation (4)) of ground-state *n*-acene in the continuum solvent, obtained from spin-unrestricted TAO-PCM (i.e., TAO-LDA/C-PCM). Note that the $S_{\mathrm{vN}}$ value is close to 0 for an electronic system with single-reference character (i.e., all the TOONs are very close to either 0 or 1), and can be much larger than 0 for an electronic system with significant MR character (i.e., some of the TOONs considerably deviate from the values of 0 and 1).

**Figure 5:**
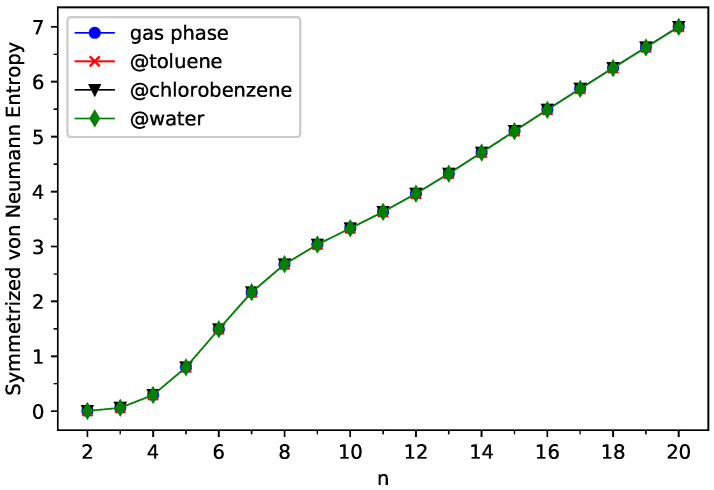
Symmetrized von Neumann entropy for the ground state of *n*-acene in three different solvents (toluene, chlorobenzene, and water) and in the gas phase, obtained from spin-unrestricted TAO-PCM (i.e., TAO-LDA/C-PCM).

As shown in Figure 5 (also see Table S6 in the SI for additional information), the $S_{\mathrm{vN}}$ value of ground-state *n*-acene in the continuum solvent increases with *n*, implying that the respective strength of MR character should also increase with *n*. In addition, the $S_{\mathrm{vN}}$ values of ground-state *n*-acenes in three different solvents (toluene, chlorobenzene, and water) are essentially identical to those of ground-state *n*-acenes in the gas phase [38,39,43,45], suggesting that the solvation effects are unimportant in affecting the $S_{\mathrm{vN}}$ values (i.e., a measure of the strength of MR character) of ground-state *n*-acenes.

### 4.4. Active TAO-Orbital Occupation Numbers

In order to see why the aforementioned $S_{\mathrm{vN}}$ value increases with *n*, we reveal the active TOONs of ground-state *n*-acene (with *N* electrons) in the continuum solvent, obtained with spin-restricted TAO-PCM (i.e., TAO-LDA/C-PCM). The ${\left(N/2\right)}^{\mathrm{th}}$/${\left(N/2+1\right)}^{\mathrm{th}}$ TAO-orbital is denoted as the HOMO (highest occupied molecular orbital)/LUMO (lowest unoccupied molecular orbital) as convention. In addition, the TAO-orbitals with an occupation number in the range of 0.2–1.8 are considered as the active TAO-orbitals.

**Figure 6:**
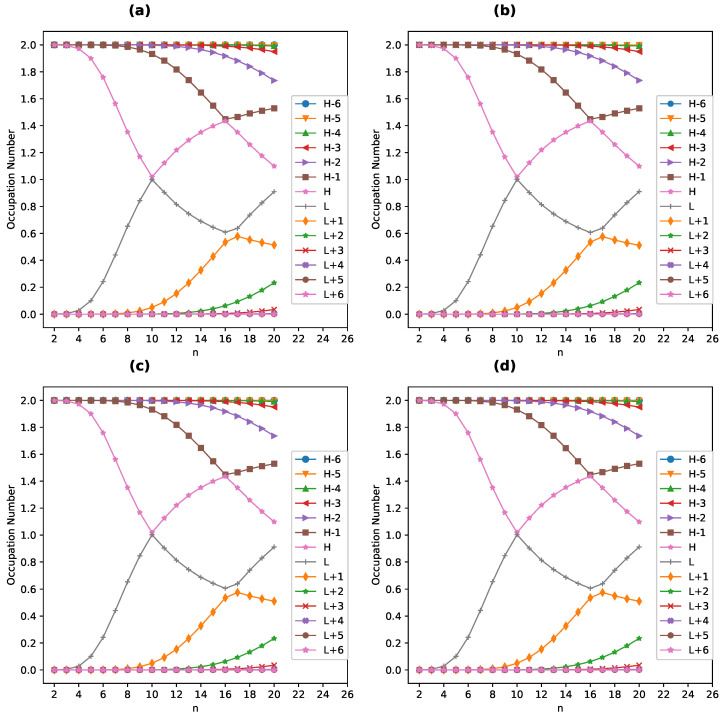
Occupation numbers of active TAO-orbitals for the ground state of *n*-acene in (**a**) the gas phase and in three different solvents: (**b**) toluene, (**c**) chlorobenzene, and (**d**) water, obtained from spin-restricted TAO-PCM (i.e., TAO-LDA/C-PCM). For brevity, the H/L is used for the HOMO/LUMO.

As plotted in Figure 6, the active TOONs of ground-state *n*-acene in the continuum solvent possess interesting patterns. First, the smaller *n*-acenes (e.g., n<6) exhibit a nonradical nature since all the TOONs are in the vicinity of either 0 or 2. Second, as the size of *n*-acene increases, there are more active TAO-orbitals and/or the active TOONs are closer to 1, displaying that the larger ground-state *n*-acenes in the continuum solvent exhibit an increasing polyradical nature. Therefore, there is a transition from a nonradical nature to polyradical nature of ground-state *n*-acene in the continuum solvent, causing an increase in $S_{\mathrm{vN}}$ with *n*. In addition, the active TOONs of ground-state *n*-acenes in three solvents (toluene, chlorobenzene, and water) are essentially the same as those of ground-state *n*-acenes in the gas phase (i.e., including the previously observed curve-crossing behavior [37,39], which was recently confirmed by an accurate MR method [31]) [37,39,43,44,45], indicating that the solvation effects are indeed insignificant in changing the MR nature of ground-state *n*-acenes (i.e., showing consistency with the $S_{\mathrm{vN}}$ analysis).

### 4.5. Active TAO-Orbitals in Real Space

In real space, we reveal the active TAO-orbitals (see Figures S1–S3 in the SI), such as the HOMO and LUMO, for the ground states of some representative *n*-acenes (e.g., 4-acene, 6-acene, and 8-acene) in the continuum solvent, obtained with spin-restricted TAO-PCM (i.e., TAO-LDA/C-PCM). As *n* increases, the active TAO-orbitals exhibit an increasing trend to localize near the edges of *n*-acene. In addition, the active TAO-orbitals of ground-state *n*-acenes in three solvents (toluene, chlorobenzene, and water) and in the gas phase [45] look very much alike, suggesting that the solvation effects play unimportant roles here.

## 5. Conclusions

In conclusion, we developed TAO-PCM, combining TAO-DFT with the PCM, to study solvation effects on the GS properties of nanomolecules with MR character at a minimal computational cost. In addition, we adopted TAO-PCM (i.e., TAO-LDA/C-PCM) to explore the electronic properties (the ST gap, fundamental gap, vertical electron affinity/ionization potential, etc.) of *n*-acene (i.e., the solute) with *n* = 2–20 in three different solvents (toluene, chlorobenzene, and water) and in the gas phase. For comparison, our TAO-PCM results (e.g., the ST gap and $E_{\mathrm{UR}}$ value of *n*-acene) were also compared with the corresponding KS-PCM (i.e., KS-LDA/C-PCM) results.

In the three solvents and in the gas phase, since the larger *n*-acenes have been found to reveal MR character in the lowest singlet states (i.e., ground states), KS-PCM can yield incorrect electronic properties (e.g., those that depend on the spin densities). For example, the ground states of some larger *n*-acenes in different media, obtained from spin-unrestricted KS-PCM, have been shown to be highly spin-contaminated, yielding unphysical spin-symmetry-breaking effects. While accurate MR methods may remedy this issue, these MR methods and related PCMs could be computationally impossible for the larger *n*-acenes in the gas phase and solution phase, respectively. Hence, it is well justified to use TAO-PCM to explore the electronic properties of *n*-acenes in different media because of its decent compromise between accuracy and efficiency.

According to TAO-PCM, in the three solvents, the smaller *n*-acenes (e.g., n<6) reveal a nonradical nature, and the larger *n*-acenes reveal an increasing polyradical nature, similar to the previous findings in the gas-phase situations [37,38,39,43,44,45]. In addition, significant changes in some of the electronic properties (e.g., the fundamental gap and vertical electron affinity/ionization potential) of ground-state *n*-acene have been found due to the presence of these solvents, highlighting the importance of TAO-PCM in the modeling of solvent effects on the GS properties of nanomolecules with MR character. Among all the electronic properties studied, the solvent polarity does not have a significant impact when the net charge is not varied in the process (e.g., the singlet–triplet gap, symmetrized von Neumann entropy, active TOONs, etc.), and has a significant impact when the net charge is varied in the process (e.g., the vertical electron affinity/ionization potential).

While TAO-PCM seems to be promising for the study of solvation effects on the GS properties of nanomolecules with MR character, TAO-PCM may not work well in situations where the underlying PCM (or other implicit solvation models) could fail badly. Therefore, in the near future, we plan to explore the possibility of combining TAO-DFT with an efficient explicit solvent model to resolve this issue.

## Figures and Tables

**Table 1:** $\langle {\hat{S}}^{2}\rangle$ for the lowest singlet state of *n*-acene in three different solvents (toluene, chlorobenzene, and water) and in the gas phase, calculated using spin-unrestricted KS-PCM (i.e., KS-LDA/C-PCM). For the lowest singlet state, the exact $\langle {\hat{S}}^{2}\rangle$ is 0.

*n*	Gas Phase	Toluene	Chlorobenzene	Water
2	0.0000	0.0000	0.0000	0.0000
3	0.0000	0.0000	0.0000	0.0000
4	0.0000	0.0000	0.0000	0.0000
5	0.0000	0.0000	0.0000	0.0000
6	0.0000	0.0000	0.0000	0.0000
7	0.0001	0.0001	0.0001	0.0001
8	0.0233	0.0298	0.0307	0.0024
9	0.8549	0.8537	0.8542	0.8563
10	1.0391	1.0389	1.0388	1.0388
11	0.9574	0.9546	0.9529	0.9514
12	0.7481	0.7415	0.7378	0.7336
13	0.0007	0.4700	0.4642	0.4602
14	0.0006	0.0005	0.0007	0.0005
15	0.0007	0.0003	0.0004	0.0012
16	0.0014	0.0007	0.0008	0.0173
17	0.0026	0.0478	0.0463	0.0584
18	0.0004	0.0007	0.0009	0.0009
19	0.8978	0.0002	0.7472	0.8890
20	1.1886	0.0008	1.1840	1.1831

**Table 2:** $\langle {\hat{S}}^{2}\rangle$ for the lowest triplet state of *n*-acene in three different solvents (toluene, chlorobenzene, and water) and in the gas phase, calculated using spin-unrestricted KS-PCM (i.e., KS-LDA/C-PCM). For the lowest triplet state, the exact $\langle {\hat{S}}^{2}\rangle$ is 2.

*n*	Gas Phase	Toluene	Chlorobenzene	Water
2	2.0053	2.0052	2.0052	2.0052
3	2.0045	2.0045	2.0045	2.0045
4	2.0047	2.0047	2.0047	2.0047
5	2.0049	2.0048	2.0048	2.0048
6	2.0050	2.0050	2.0050	2.0049
7	2.0051	2.0051	2.0051	2.0051
8	2.0052	2.0052	2.0052	2.0052
9	2.0053	2.0053	2.0052	2.0052
10	2.0053	2.0053	2.0053	2.0053
11	2.0053	2.0053	2.0053	2.0053
12	2.0053	2.0053	2.0053	2.0053
13	2.0053	2.0053	2.0052	2.0052
14	2.0052	2.0052	2.0052	2.0052
15	2.0206	2.0206	2.0051	2.0207
16	2.0315	2.0312	2.0310	2.0309
17	2.0230	2.0226	2.0223	2.0220
18	2.0335	2.0331	2.0329	2.0327
19	2.0372	2.0366	2.0364	2.0362
20	2.0412	2.0406	2.0404	2.0402

**Table 3:** Difference $E_{\mathrm{UR}}$ (in kcal/mol) between the lowest spin-unrestricted and lowest spin-restricted singlet free energies of *n*-acene in the gas phase and in three different solvents (toluene, chlorobenzene, and water), calculated using KS-PCM (i.e., KS-LDA/C-PCM).

*n*	Gas Phase	Toluene	Chlorobenzene	Water
2	0.00	0.00	0.00	0.00
3	0.00	0.00	0.00	0.00
4	0.00	0.00	0.00	0.00
5	0.00	0.00	0.00	0.00
6	0.00	0.00	0.00	0.00
7	0.00	0.00	0.00	0.00
8	0.00	0.00	0.00	0.00
9	0.93	0.93	0.93	0.93
10	3.26	3.23	3.21	3.20
11	1.58	1.55	1.54	1.53
12	0.66	0.64	0.63	0.62
13	0.00	0.19	0.19	0.18
14	0.00	0.00	0.00	0.00
15	0.00	0.00	0.00	0.00
16	0.00	0.00	0.00	0.00
17	0.00	0.00	0.00	0.00
18	0.00	0.00	0.00	0.00
19	0.43	0.00	0.34	0.42
20	1.09	0.00	1.08	1.08

## Data Availability

The numerical data supporting the findings of the present work are available from the authors upon appropriate request.

## Supplementary Materials

The following supporting information can be downloaded at: https://www.mdpi.com/article/10.3390/nano13101593/s1, Figure S1: Real-space representation of active TAO-orbitals (HOMO and LUMO) for the ground state of 4-acene in (a) the gas phase and in three different solvents: (b) toluene, (c) chlorobenzene, and (d) water, calculated using spin-restricted TAO-PCM (i.e., TAO-LDA/C-PCM), at an isovalue of 0.02 e/Å${}^{3}$; Figure S2: Real-space representation of active TAO-orbitals (HOMO and LUMO) for the ground state of 6-acene in (a) the gas phase and in three different solvents: (b) toluene, (c) chlorobenzene, and (d) water, calculated using spin-restricted TAO-PCM (i.e., TAO-LDA/C-PCM), at an isovalue of 0.02 e/Å${}^{3}$; Figure S3: Real-space representation of active TAO-orbitals (HOMO and LUMO) for the ground state of 8-acene in (a) the gas phase and in three different solvents: (b) toluene, (c) chlorobenzene, and (d) water, calculated using spin-restricted TAO-PCM (i.e., TAO-LDA/C-PCM), at an isovalue of 0.02 e/Å${}^{3}$; Table S1: Singlet-triplet energy gap $E_{\mathrm{ST}}$ (in kcal/mol) of *n*-acene in the gas phase and in three different solvents (toluene, chlorobenzene, and water), calculated using spin-unrestricted TAO-PCM (i.e., TAO-LDA/C-PCM); Table S2: Singlet-triplet energy gap $E_{\mathrm{ST}}$ (in kcal/mol) of *n*-acene in the gas phase and in three different solvents (toluene, chlorobenzene, and water), calculated using spin-unrestricted KS-PCM (i.e., KS-LDA/C-PCM); Table S3: Vertical ionization potential ${\mathrm{IP}}_{v}$ (in eV) for the ground state of *n*-acene in the gas phase and in three different solvents (toluene, chlorobenzene, and water), calculated using spin-unrestricted TAO-PCM (i.e., TAO-LDA/C-PCM); Table S4: Vertical electron affinity ${\mathrm{EA}}_{v}$ (in eV) for the ground state of *n*-acene in the gas phase and in three different solvents (toluene, chlorobenzene, and water), calculated using spin-unrestricted TAO-PCM (i.e., TAO-LDA/C-PCM); Table S5: Fundamental gap $E_{g}$ (in eV) for the ground state of *n*-acene in the gas phase and in three different solvents (toluene, chlorobenzene, and water), calculated using spin-unrestricted TAO-PCM (i.e., TAO-LDA/C-PCM); Table S6: Symmetrized von Neumann entropy $S_{\mathrm{vN}}$ for the ground state of *n*-acene in the gas phase and in three different solvents (toluene, chlorobenzene, and water), calculated using spin-unrestricted TAO-PCM (i.e., TAO-LDA/C-PCM).
