# Supplementary material for: TAO-DFT with the Polarizable Continuum Model
Source: Nanomaterials (Basel). 2023 May 10;13(10):1593. doi: 10.3390/nano13101593 (PMC10222483; doi:10.3390/nano13101593)
Supplement: Supplementary file 1 [file nanomaterials-13-01593-s001.zip › nanomaterials-2384954-supplementary.pdf]

Supplementary Information to: TAO-DFT with the Polarizable Continuum Model

Sonai Seenithurai[1] and Jeng-Da Chai[1,2,3,*]

[1]*Department of Physics, National Taiwan University, Taipei 10617, Taiwan*

[2]*Center for Theoretical Physics and Center for Quantum Science and Engineering, National Taiwan University, Taipei 10617, Taiwan*

[3]*Physics Division, National Center for Theoretical Sciences, Taipei 10617, Taiwan*

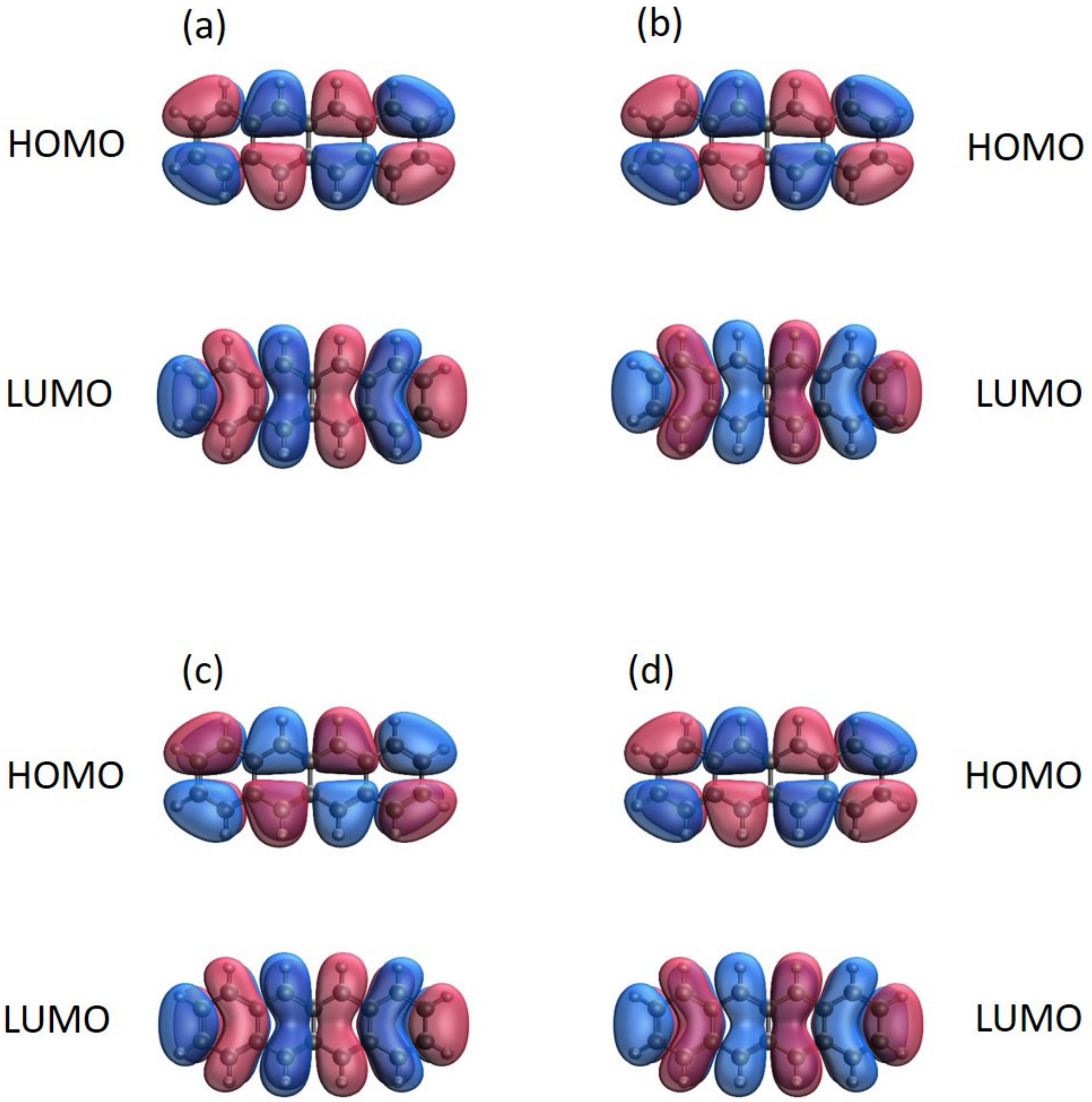

FIG. S1. Real-space representation of active TAO-orbitals (HOMO and LUMO) for the ground state of 4-acene in (a) the gas phase and in three different solvents: (b) toluene, (c) chlorobenzene, and (d) water, calculated using spin-restricted TAO-PCM (i.e., TAO-LDA / C-PCM), at an isovalue of 0.02 e/Å^3.

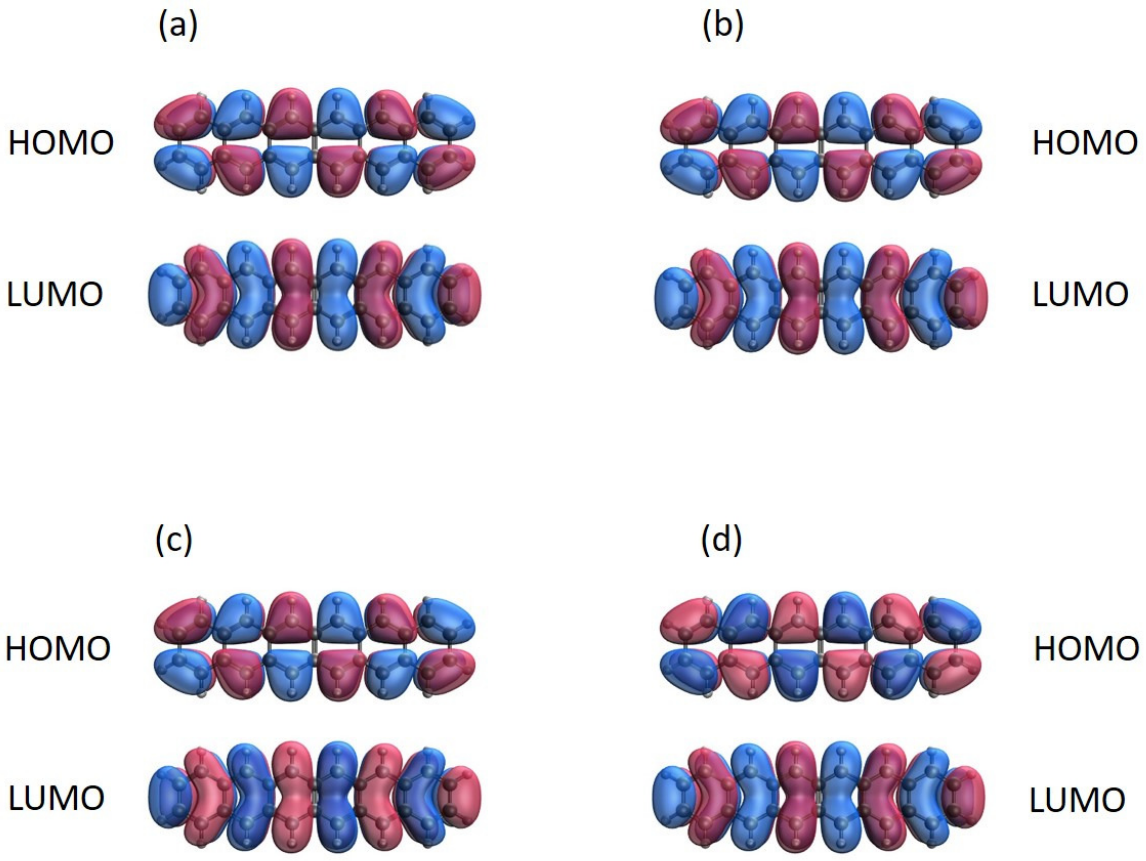

FIG. S2. Real-space representation of active TAO-orbitals (HOMO and LUMO) for the ground state of 6-acene in (a) the gas phase and in three different solvents: (b) toluene, (c) chlorobenzene, and (d) water, calculated using spin-restricted TAO-PCM (i.e., TAO-LDA / C-PCM), at an isovalue of 0.02 e/Å^3.

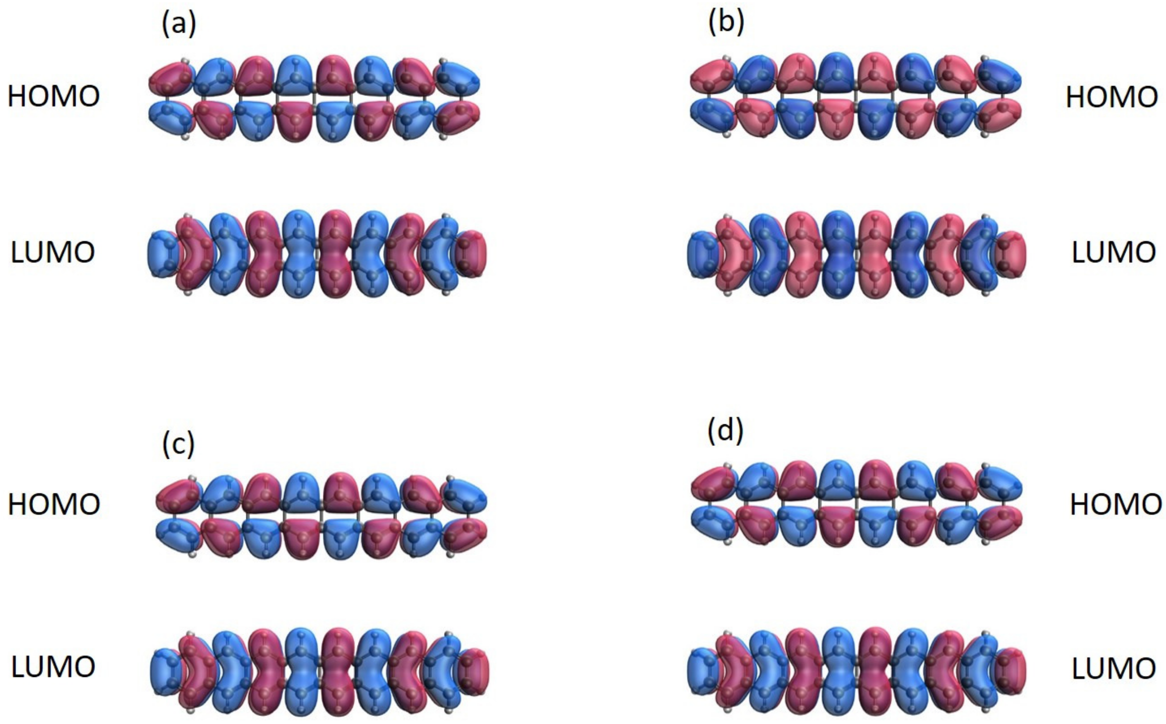

FIG. S3. Real-space representation of active TAO-orbitals (HOMO and LUMO) for the ground state of 8-acene in (a) the gas phase and in three different solvents: (b) toluene, (c) chlorobenzene, and (d) water, calculated using spin-restricted TAO-PCM (i.e., TAO-LDA / C-PCM), at an isovalue of 0.02 e/Å^3.

TABLE S1. Singlet-triplet energy gap E_{ST} (in kcal/mol) of n-acene in the gas phase and in three different solvents (toluene, chlorobenzene, and water), calculated using spin-unrestricted TAO-PCM (i.e., TAO-LDA / C-PCM).

n	Gas Phase	Toluene	Chlorobenzene	Water
2	64.77	64.81	64.83	64.84
3	43.22	43.25	43.26	43.27
4	29.01	29.04	29.05	29.05
5	19.60	19.61	19.62	19.62
6	13.55	13.56	13.56	13.56
7	9.91	9.92	9.92	9.92
8	7.84	7.85	7.85	7.86
9	6.66	6.67	6.67	6.68
10	5.91	5.91	5.92	5.92
11	5.32	5.33	5.34	5.34
12	4.82	4.83	4.83	4.84
13	4.38	4.38	4.39	4.39
14	3.98	3.99	3.99	4.00
15	3.65	3.66	3.66	3.66
16	3.37	3.38	3.38	3.38
17	3.14	3.15	3.15	3.15
18	2.94	2.95	2.95	2.95
19	2.77	2.78	2.78	2.78
20	2.62	2.63	2.63	2.63

TABLE S2. Singlet-triplet energy gap E_{ST} (in kcal/mol) of n-acene in the gas phase and in three different solvents (toluene, chlorobenzene, and water), calculated using spin-unrestricted KS-PCM (i.e., KS-LDA / C-PCM).

n	Gas Phase	Toluene	Chlorobenzene	Water
2	65.19	65.23	65.25	65.26
3	43.40	43.43	43.44	43.45
4	29.06	29.08	29.09	29.10
5	19.20	19.22	19.22	19.23
6	12.16	12.17	12.17	12.17
7	6.98	6.98	6.98	6.98
8	3.07	3.07	3.07	3.06
9	0.99	0.98	0.98	0.98
10	0.53	0.53	0.53	0.52
11	0.86	0.87	0.87	0.87
12	1.59	1.61	1.61	1.62
13	2.31	2.34	2.36	2.55
14	3.47	3.52	3.51	3.53
15	4.34	4.36	4.49	4.39
16	4.43	4.45	4.46	4.47
17	3.90	3.91	3.92	3.92
18	3.01	3.00	3.00	2.99
19	1.96	1.52	1.85	1.93
20	1.30	1.28	1.28	1.27

TABLE S3. Vertical ionization potential IP_v (in eV) for the ground state of n-acene in the gas phase and in three different solvents (toluene, chlorobenzene, and water), calculated using spin-unrestricted TAO-PCM (i.e., TAO-LDA / C-PCM).

n	Gas Phase	Toluene	Chlorobenzene	Water
2	7.81	6.67	6.20	5.88
3	7.00	6.00	5.58	5.30
4	6.46	5.55	5.17	4.91
5	6.07	5.23	4.89	4.65
6	5.79	5.01	4.69	4.47
7	5.59	4.86	4.56	4.36
8	5.44	4.76	4.48	4.29
9	5.33	4.69	4.42	4.24
10	5.23	4.63	4.38	4.21
11	5.15	4.58	4.34	4.18
12	5.08	4.53	4.31	4.15
13	5.01	4.49	4.28	4.13
14	4.96	4.46	4.25	4.11
15	4.91	4.43	4.23	4.10
16	4.86	4.40	4.21	4.08
17	4.82	4.37	4.19	4.07
18	4.78	4.35	4.18	4.06
19	4.75	4.33	4.16	4.05
20	4.71	4.31	4.15	4.04

TABLE S4. Vertical electron affinity EA_v (in eV) for the ground state of n-acene in the gas phase and in three different solvents (toluene, chlorobenzene, and water), calculated using spin-unrestricted TAO-PCM (i.e., TAO-LDA / C-PCM).

n	Gas Phase	Toluene	Chlorobenzene	Water
2	-0.62	0.59	1.10	1.46
3	0.28	1.36	1.82	2.15
4	0.90	1.88	2.31	2.60
5	1.34	2.25	2.64	2.92
6	1.66	2.50	2.87	3.13
7	1.89	2.68	3.03	3.28
8	2.06	2.81	3.14	3.37
9	2.19	2.90	3.21	3.43
10	2.30	2.97	3.26	3.48
11	2.39	3.03	3.31	3.51
12	2.48	3.08	3.35	3.54
13	2.55	3.13	3.39	3.57
14	2.61	3.17	3.42	3.59
15	2.67	3.21	3.44	3.61
16	2.72	3.24	3.47	3.63
17	2.77	3.27	3.49	3.65
18	2.81	3.29	3.51	3.66
19	2.85	3.32	3.52	3.67
20	2.89	3.34	3.54	3.68

TABLE S5. Fundamental gap E_g (in eV) for the ground state of n-acene in the gas phase and in three different solvents (toluene, chlorobenzene, and water), calculated using spin-unrestricted TAO-PCM (i.e., TAO-LDA / C-PCM).

n	Gas Phase	Toluene	Chlorobenzene	Water
2	8.43	6.08	5.10	4.42
3	6.73	4.64	3.76	3.16
4	5.56	3.67	2.86	2.31
5	4.73	2.98	2.24	1.73
6	4.13	2.51	1.82	1.34
7	3.69	2.18	1.53	1.08
8	3.38	1.95	1.35	0.92
9	3.13	1.79	1.21	0.81
10	2.93	1.66	1.11	0.73
11	2.76	1.55	1.03	0.67
12	2.60	1.45	0.96	0.61
13	2.46	1.36	0.89	0.56
14	2.34	1.29	0.83	0.52
15	2.23	1.22	0.78	0.48
16	2.13	1.16	0.74	0.45
17	2.05	1.10	0.70	0.42
18	1.97	1.06	0.67	0.40
19	1.89	1.01	0.64	0.38
20	1.83	0.97	0.61	0.36

TABLE S6. Symmetrized von Neumann entropy S_{vN} for the ground state of n-acene in the gas phase and in three different solvents (toluene, chlorobenzene, and water), calculated using spin-unrestricted TAO-PCM (i.e., TAO-LDA / C-PCM).

n	Gas Phase	Toluene	Chlorobenzene	Water
2	0.00	0.00	0.00	0.00
3	0.06	0.06	0.06	0.06
4	0.30	0.30	0.30	0.29
5	0.80	0.80	0.80	0.80
6	1.49	1.49	1.49	1.49
7	2.17	2.17	2.17	2.17
8	2.68	2.68	2.68	2.68
9	3.03	3.03	3.04	3.04
10	3.33	3.33	3.33	3.33
11	3.63	3.63	3.63	3.63
12	3.96	3.96	3.96	3.96
13	4.33	4.33	4.33	4.33
14	4.71	4.71	4.71	4.71
15	5.10	5.10	5.10	5.10
16	5.49	5.49	5.49	5.49
17	5.88	5.87	5.87	5.87
18	6.25	6.25	6.25	6.25
19	6.63	6.63	6.63	6.62
20	7.01	7.00	7.00	7.00
